# Mitofusins: Disease Gatekeepers and Hubs in Mitochondrial Quality Control by E3 Ligases

**DOI:** 10.3389/fphys.2019.00517

**Published:** 2019-05-09

**Authors:** Mafalda Escobar-Henriques, Mariana Joaquim

**Affiliations:** Center for Molecular Medicine Cologne (CMMC), Institute for Genetics, Cologne Excellence Cluster on Cellular Stress Responses in Aging-Associated Diseases (CECAD), University of Cologne, Cologne, Germany

**Keywords:** E3 ligases, ubiquitin, mitofusins, MFN1/MFN2, mitochondria, quality control, mitophagy, ER

## Abstract

Mitochondria are dynamic organelles engaged in quality control and aging processes. They constantly undergo fusion, fission, transport, and anchoring events, which empower mitochondria with a very interactive behavior. The membrane remodeling processes needed for fusion require conserved proteins named mitofusins, MFN1 and MFN2 in mammals and Fzo1 in yeast. They are the first determinants deciding on whether communication and content exchange between different mitochondrial populations should occur. Importantly, each cell possesses hundreds of mitochondria, with a different severity of mitochondrial mutations or dysfunctional proteins, which potentially spread damage to the entire network. Therefore, the degree of their merging capacity critically influences cellular fitness. In turn, the mitochondrial network rapidly and dramatically changes in response to metabolic and environmental cues. Notably, cancer or obesity conditions, and stress experienced by neurons and cardiomyocytes, for example, triggers the downregulation of mitofusins and thus fragmentation of mitochondria. This places mitofusins upfront in sensing and transmitting stress. In fact, mitofusins are almost entirely exposed to the cytoplasm, a topology suitable for a critical relay point in information exchange between mitochondria and their cellular environment. Consistent with their topology, mitofusins are either activated or repressed by cytosolic post-translational modifiers, mainly by ubiquitin. Ubiquitin is a ubiquitous small protein orchestrating multiple quality control pathways, which is covalently attached to lysine residues in its substrates, or in ubiquitin itself. Importantly, from a chain of events also mediated by E1 and E2 enzymes, E3 ligases perform the ultimate and determinant step in substrate choice. Here, we review the ubiquitin E3 ligases that modify mitofusins. Two mitochondrial E3 enzymes—March5 and MUL1—one ligase located to the ER—Gp78—and finally three cytosolic enzymes—MGRN1, HUWE1, and Parkin—were shown to ubiquitylate mitofusins, in response to a variety of cellular inputs. The respective outcomes on mitochondrial morphology, on contact sites to the endoplasmic reticulum and on destructive processes, like mitophagy or apoptosis, are presented. Ultimately, understanding the mechanisms by which E3 ligases and mitofusins sense and bi-directionally signal mitochondria-cytosolic dysfunctions could pave the way for therapeutic approaches in neurodegenerative, cardiovascular, and obesity-linked diseases.

## Introduction

Mitochondria were considered as static and isolated bean-shaped organelles for a long time, being labeled “power house of the cell” given the assumption that ATP production by oxidative phosphorylation (OXPHOS) was their main function (Mcbride and Neuspiel, 2006). However, as soon as researchers started to look into it by live imaging, it was quickly perceptible the existence of a high dynamism (Bereiter-Hahn and Voth, 1994; Nunnari et al., 1997), later proved to be associated with many new mitochondrial functions (Westermann, 2010). Mitochondria possess proteins that enable plastic responses, depending on the cellular conditions, by fusion, fission, and transport processes (Friedman and Nunnari, 2014). Another hallmark in the field was the awareness of the importance of mitochondrial transport and positioning within the cell and thereby interaction with other cellular compartments surrounding it. Pioneering studies unraveling physical tethering between mitochondria and the endoplasmic reticulum (ER; Kornmann et al., 2009) paved the way for subsequent discoveries on several other mitochondrial contact sites (Eisenberg-bord and Schuldiner, 2017; Cohen et al., 2018). These contacts coordinate a continuous communication of mitochondria with other organelles to support important cellular functions. Finally, the functional impact of mitochondrial interaction with soluble components present in the cytoplasm, like ubiquitin, revealed another layer of the integrative behavior of these organelles (Escobar-Henriques and Langer, 2014; Bragoszewski et al., 2017).

Ubiquitylation is a post-translational modification (PTM) that occurs through the addition of a ubiquitin moiety to substrates (Yau and Rape, 2016; Kwon and Ciechanover, 2017). Ubiquitin is required for many cellular pathways, and the discovery of its regulatory functions is constantly increasing (Rape, 2018). Consistently, ubiquitin targets at mitochondria are associated with several distinct and important cellular processes, mainly with cellular quality control functions (Escobar-Henriques and Langer, 2014).

Here we present the current knowledge on the E3 ligases modifying mitochondrial proteins, focusing on the mitochondrial fusion factors Mitofusin 1 (MFN1) and Mitofusin 2 (MFN2). Mitofusins appear to be preferred targets, constituting a cellular hub in response to metabolic needs of the cell (Chan et al., 2011; Sarraf et al., 2013; Bingol et al., 2014).

### Ubiquitylation

Ubiquitylation of proteins is one of the cellular PTMs, which allow diversifying the coding capacity of genes by covalent modifications, mostly enzyme-catalyzed, of nascent or folded proteins. Therefore, PTMs create a bigger pool of protein diversity (Walsh et al., 2005). The most common small PTMs are phosphorylation, acetylation, glycosylation, carboxylation, methylation, nitrosylation, and S-glycation, which are characterized by the addition of the respective chemical moieties to proteins (Walsh et al., 2005). Moreover, ubiquitin and ubiquitin-like modifiers constitute a set of additional PTMs: ubiquitylation, sumoylation, rubylation, lipidation, ISGylation, and FATylation (Cappadocia and Lima, 2017). Interestingly, ubiquitin itself was shown to be post-translationally modified by phosphorylation and acetylation (Herhaus and Dikic, 2015; Swatek and Komander, 2016).

**Figure 1:**
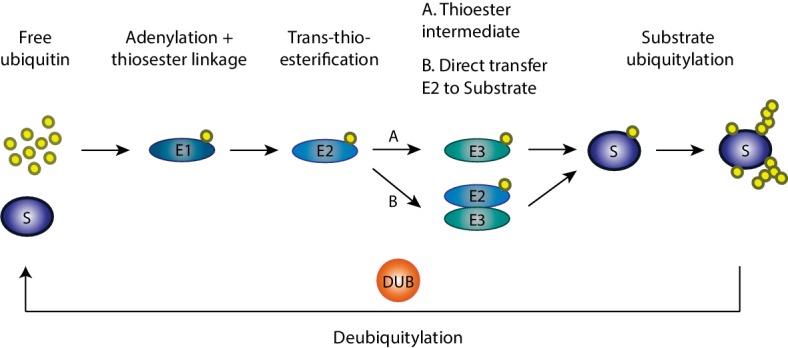
Ubiquitylation cascade. Ubiquitylation of substrates requires a cascade of events involving three enzymes: an E1 ubiquitin-activating enzyme, an E2 ubiquitin-conjugating enzyme, and an E3 ubiquitin ligase. First in this cascade, the E1 enzyme activates ubiquitin and transfers it to the E2 enzyme in an ATP-dependent manner with which ubiquitin is conjugated. Afterward, the ubiquitin molecule is transferred from the E2 enzyme to the specific target substrate by the E3 ligase enzymes, which either actively receives ubiquitin from E2 and then transfers it to the substrate or serves as a binding platform between the E2 and the substrate. Finally, on the target substrate, mono, mono-multi, or polyubiquitylation can occur.

Ubiquitin is a small highly conserved eukaryotic protein and ubiquitylation is the process by which ubiquitin molecules are added to a substrate (Ciechanover, 2005) (Figure 1). It occurs *via* an enzymatic cascade involving three elements: an E1 ubiquitin-activating enzyme, an E2 ubiquitin-conjugating enzyme, and an E3 ubiquitin ligase. First, the E1 enzyme activates ubiquitin and transfers it to the E2 enzyme, in an ATP-dependent manner. Subsequently, the ubiquitin molecule is transferred from the E2 enzyme to a specific target substrate. This requires substrate recognition by an E3 ligase, which either actively receives ubiquitin from the E2 and then covalently binds it to the substrate (HECT, RBR) or serves as a binding platform between the E2 and the substrate (RING) (Komander and Rape, 2012; Yau and Rape, 2016). E3 ligases are of extreme importance in this enzymatic cascade, since they select the specific substrates to be modified (Zheng and Shabek, 2017). Importantly, ubiquitylation is a reversible process, where deubiquitylases are able to remove the ubiquitin moiety from a substrate, resulting in free ubiquitin (Mevissen and Komander, 2017; Clague et al., 2019). Ubiquitin can be present in substrates in the form of one ubiquitin moiety (mono-ubiquitylation) or several moieties (multi-monoubiquitylation). Moreover, poly-ubiquitin chains of different topologies can also form, *via* any of the seven internal lysine residues in ubiquitin (Lys6, Lys11, Lys27, Lys29, Lys33, Lys48, and Lys63; Komander and Rape, 2012; Yau and Rape, 2016). Due to their different surfaces, these ubiquitin chains attract diverse effectors, giving origin to a variety of functions (Kwon and Ciechanover, 2017). For example, Lys48-linked chains are mostly known to mark proteins for proteasomal degradation *via* the ubiquitin-proteasome system (UPS), whereas Lys63-linked chains are mainly associated with regulatory functions (Kwon and Ciechanover, 2017).

### Mitochondria and Mitofusins

**Figure 2:**
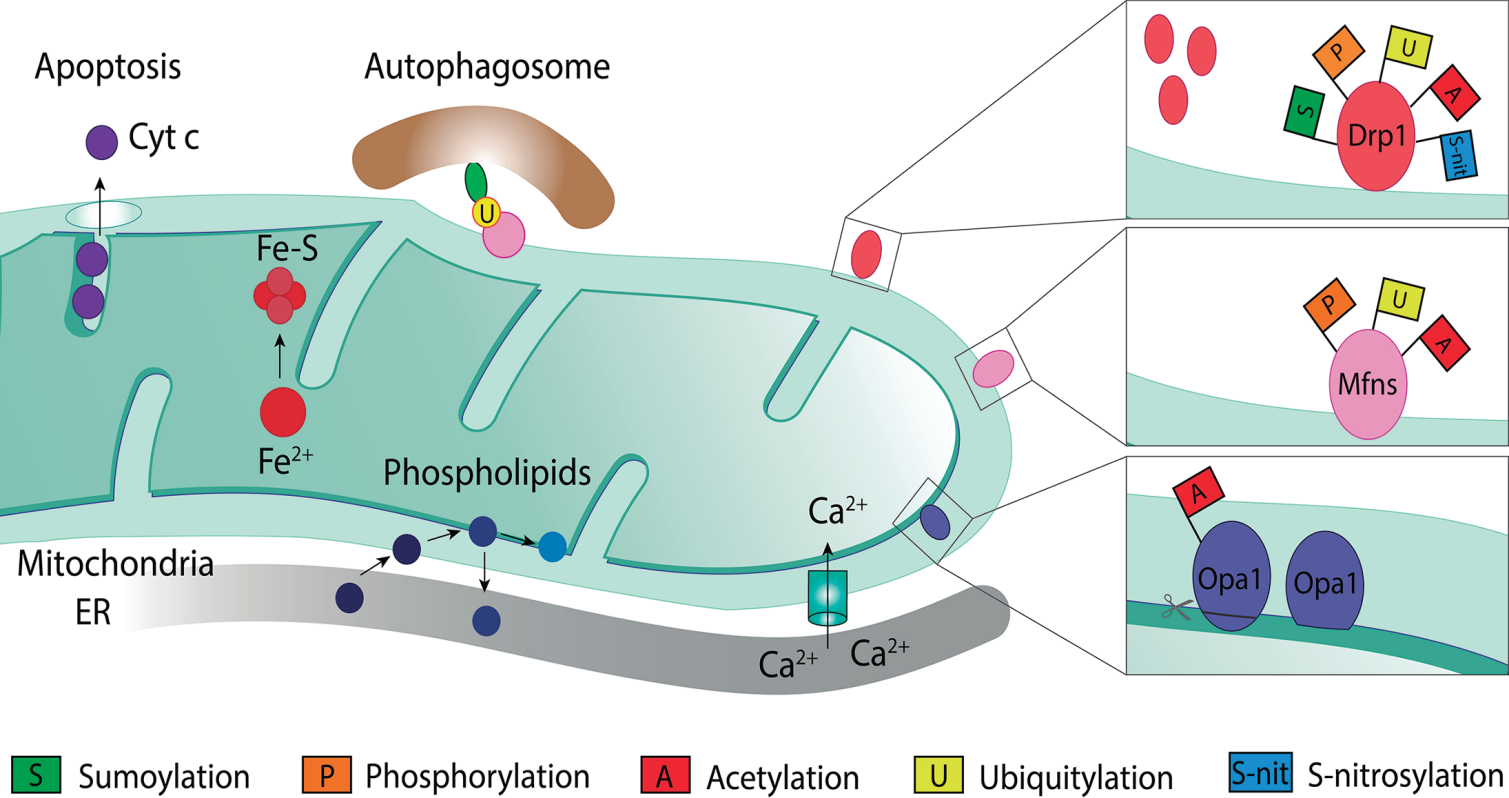
Mitochondrial roles and post-translational modifications of mitochondrial dynamic proteins. Mitochondria are involved in several cellular processes other than ATP production, such as calcium (Ca^2+^) buffering, phospholipid biosynthesis, iron-sulfur clusters (Fe-S) assembly from iron (Fe^2+^), regulation of programmed cell death *via* cytochrome c (Cytc c) release, and mitophagy. Mitochondrial proteins known for their role in mitochondrial dynamics, such as Drp1, Mfn1/2, and Opa1, are a target for several post-translational modifications, which differently regulate their level and, hence, function. Drp1, on the OMM, can be modified by sumoylation, phosphorylation, acetylation, ubiquitylation, and S-nitrosylation. Mitofusins, also on the OMM, can be a target for phosphorylation, ubiquitylation, or acetylation. Opa1, in the IMM, can suffer processing or be modified through acetylation.

Mitochondria are double membrane organelles composed by the outer mitochondrial membrane (OMM) and the inner mitochondrial membrane (IMM), which are separated by the intermembrane space (IMS; Figure 2; Pfanner et al., 2019). The IMM encloses the mitochondrial matrix and forms invaginations called cristae (Frey et al., 2002). The OXPHOS system locates along the cristae and provides the mitochondrial membrane potential, necessary for the production of energy in the form of ATP (Nunnari and Suomalainen, 2012). Besides oxidative phosphorylation, mitochondria perform several other important functions, such as phospholipid synthesis and assembly of iron-sulfur clusters (Braymer and Lill, 2017; Tatsuta and Langer, 2017; Cardenas et al., 2018). In addition, mitochondria are important for cellular responses, such as calcium (Ca^2+^) buffering (Marchi et al., 2017; Xie et al., 2018), mitophagy (Harper et al., 2018; Pickles et al., 2018), and regulation of programmed cell death (Xie et al., 2018; Sedlackova and Korolchuk, 2019).

The functional plasticity of mitochondria is intimately linked to its morphology (Friedman and Nunnari, 2014; Tilokani et al., 2018). Fusion and fission events are majorly important for the regulation of mitochondrial morphology, whereas mitochondrial transport is of particular importance in cells with high-energy demands, such as neurons (Knott and Bossy-Wetzel, 2008; Rakovic et al., 2011). The dynamics between mitochondrial fusion and fission may result in several possible morphological outcomes, from a tubular mitochondrial network, sometimes massively interconnected, to several fragments. This plasticity is fundamental for the maintenance of proper mitochondrial function and to assist mitochondria in response to several stress situations (Liesa and Shirihai, 2013; Schrepfer and Scorrano, 2016; Chen and Chan, 2017). For example, loss of membrane potential and nutrient excess have been shown to induce mitochondrial fragmentation (Yu et al., 2006), whereas nutrient starvation was shown to shift the balance toward a tubular mitochondrial network (Tondera et al., 2009; Gomes et al., 2011; Rambold et al., 2011). These membrane remodeling events are mediated by conserved large dynamin-like GTPase proteins (Praefcke and McMahon, 2004). Drp1 is responsible for fission (Dnm1 in yeast), MFN1/MFN2 for OMM fusion (Fzo1 in yeast), and Opa1 for IMM fusion (Mgm1 in yeast; Youle and van der Bliek, 2012). They are main targets of PTMs, being either activated or repressed in order to push the morphology toward a fused or a fragmented state (Figure 2; Escobar-Henriques and Langer, 2014; Macvicar and Langer, 2016; Mishra and Chan, 2016).

**Figure 3:**
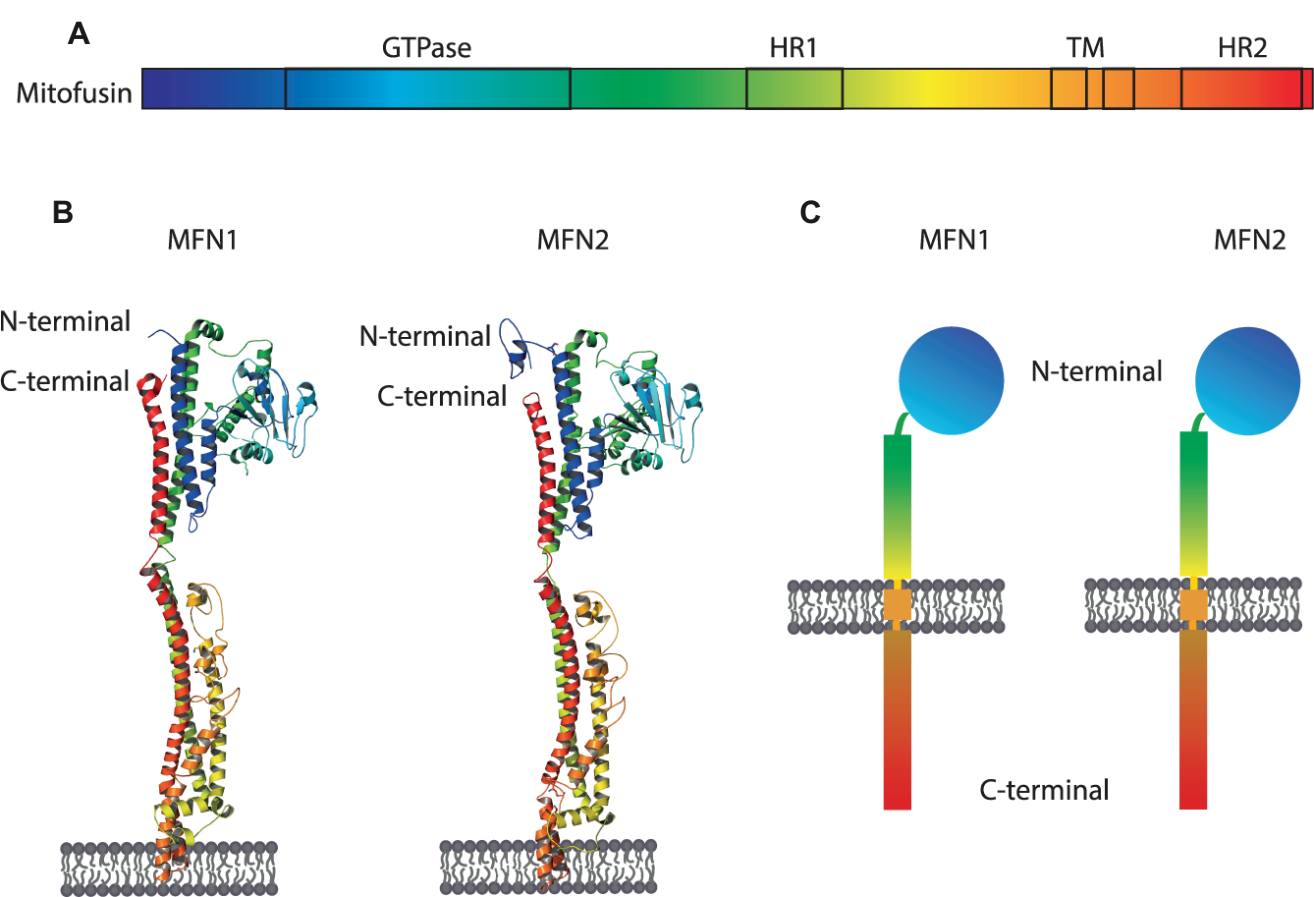
Structure and topology models of mitofusins. **(A)** Linear structure of mitofusin, with the GTPase domain locating at the N-terminal, one hydrophobic heptad repeat (HR1), the transmembrane anchor(s), and a second hydrophobic heptad repeat (HR2). **(B)** Crystal structure of MFN1 and MFN2 modeled on BDLP and mini-MFN1, according to the first topology proposed, with two transmembrane domains and both the N- and C-terminus facing the cytosol (Rojo et al., 2002; Low and Löwe, 2006; Low et al., 2009; Qi et al., 2016; Cao et al., 2017). **(C)** Structural scheme of MFN1 and MFN2 according to the second topology proposed with a single spanning-membrane domain, instead of two, and the C-terminus residing in the IMS and not facing the cytosol (Mattie et al., 2018).

**Figure 4:**
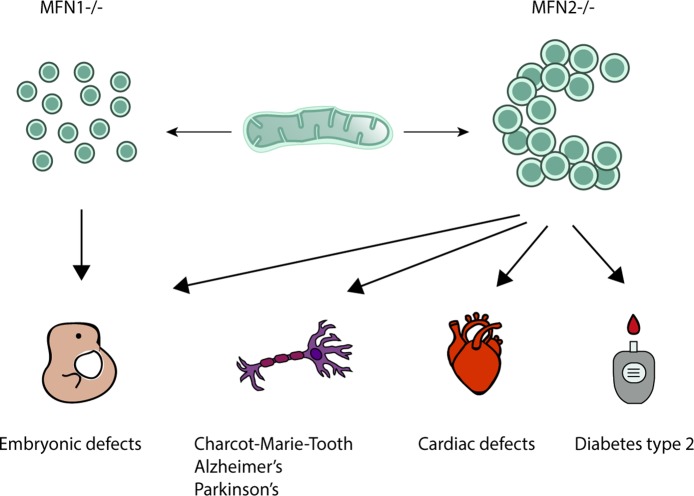
Mitochondrial morphology upon knockout of Mitofusin 1 or 2 and its disease-associated roles. Although extremely similar in their sequence and structure, each mitofusin ablation leads to strikingly different mitochondrial morphologies. Mitofusin 1 knockout gives origin to a highly fragmented mitochondrial network composed of many small fragments, whereas depletion of Mitofusin 2 leads to a network where mitochondrial fragments are found enlarged and aggregated in clusters, commonly perinuclearly organized. Several diseases have been associated with knockout of Mitofusin 1 or 2. Homozygous knockout of Mitofusin 1 or 2 leads to embryonic defects such as defective giant cell layer and leads, ultimately, to lethality. While Mitofusin 1 only appears to have an effect at the embryonic level, knockout of its homologue protein, Mitofusin 2, has been shown to relate to several other defects. Animal models depleted for Mitofusin 2 display severe cardiac defects such as cardiomyocyte dysfunction, rapid progressive dilated cardiomyopathy, and final heart failure. Moreover, Mitofusin 2 mutations are the primary cause of the incurable neuropathy Charcot-Marie Tooth Type 2A for which no disease-underlying functions have been yet identified. Additionally, links with other neurodegenerative diseases such as Alzheimer’s and Parkinson’s diseases have been made, although the molecular mechanisms underlying it are not fully understood. Low levels of Mitofusin 2 were also shown to have a strong positive correlation with diabetes type 2 and obesity.

Mitofusins are OMM proteins, with the GTPase domain locating at the N-terminal, followed by one hydrophobic heptad repeat (HR1), the transmembrane anchor(s) and finally possessing a second protein-protein interaction domain, HR2 (Figure 3). First, it was proposed that both N- and C-terminus face the cytosol, connected by two transmembrane domains and a short loop in the IMS (Rojo et al., 2002). This topology is in agreement with fusion-compatible structural information from both MFN1 and the bacterial homologue BDLP (Low and Löwe, 2006; Low et al., 2009; Qi et al., 2016; Cao et al., 2017). However, an alternative topology for MFN1 and MFN2 was proposed, with a single spanning-membrane domain, instead of two, therefore placing the C-terminus in the IMS (Mattie et al., 2018). Further studies will be necessary to elucidate which topology of mitofusins reflects fusion-dependent or perhaps fusion-independent roles of mitofusins. MFN1 and MFN2 proteins are 62% identical and 77% similar to each other (Zorzano and Pich, 2006). Interestingly, despite being extremely similar, depletion of each mitofusin has different effects on mitochondrial morphology. While depletion of MFN1 leads to highly fragmented mitochondria, in the shape of small fragments, depletion of MFN2 leads to bigger mitochondrial fragments that aggregate into clusters (Figure 4; Chen et al., 2003). Strikingly, homozygous knockout of either MFN1 or MFN2 in mice was shown to be lethal, with death occurring in midgestation (Chen et al., 2003). Additionally, MFN2 depleted mice presented placental defects within the giant cell layer, with a reduced number of giant cells and a reduced number of nuclei on the few cells still observed. However, no placental developmental defects were observed in MFN1 mutants. This suggests that MFN1 and MFN2 may have distinct functions, perhaps independent of their roles in mitochondrial fusion (Loiseau et al., 2007; Guillet et al., 2010; Codron et al., 2016; Beręsewicz et al., 2017; El Fissi et al., 2018; Zhou et al., 2019). For example, a correlation observed between the levels of MFN2 and oxidative phosphorylation was suggested to be dependent on coenzyme Q deficiency, independently of the fusion capacity of MFN2 (Pich et al., 2005; Segalés et al., 2013; Mourier et al., 2015).

## Cellular Processes Affected by Ubiquitylation of Mitofusins

Ubiquitylation of both MFN1 and MFN2 has been reported and associated with diverse cellular processes. First, responses directly affecting mitochondria themselves were described, either by changing their morphology or by extending mitochondrial contacts to the ER. Second, effects on mitophagy or apoptosis are the most described effects. Nonetheless, links of mitofusin ubiquitylation with hypoxic and genotoxic stress have also been made.

### Mitochondrial Morphology

Ubiquitylation of mitofusins is promptly observed in yeast, flies, and mammals (Cohen et al., 2008; Ziviani et al., 2010; Rakovic et al., 2011). Interestingly, it plays a dual role in mitochondrial morphology either by addressing mitofusins for proteasomal turnover or by activating mitofusins and therefore promoting membrane merging. It was first suggested that the steady-state levels of Fzo1 are regulated (Fritz et al., 2003), and it was later shown that the turnover of this protein is proteasome-dependent in response to mating factor (Neutzner and Youle, 2005). Moreover, it was shown that the AAA protein and ubiquitin-selective chaperone VCP/p97/Cdc48 is required for proteasomal-dependent degradation of mitofusins (Tanaka et al., 2010; Kim et al., 2013; Zhang et al., 2017). This results in mitochondrial fragmentation, due to ongoing fission events. Moreover, it is associated with stress responses, mediated by several E3 ligases, as outlined later. This induces different outcomes, either regulating mitochondria-ER contact sites or affecting mitophagy and apoptosis. On the other hand, pro-fusion ubiquitylation of Fzo1 occurs constitutively and is tightly controlled by deubiquitylases and Cdc48 (Anton et al., 2013; Yue et al., 2014; Simões et al., 2018).

### ER-Mitochondria Contacts

**Figure 5:**
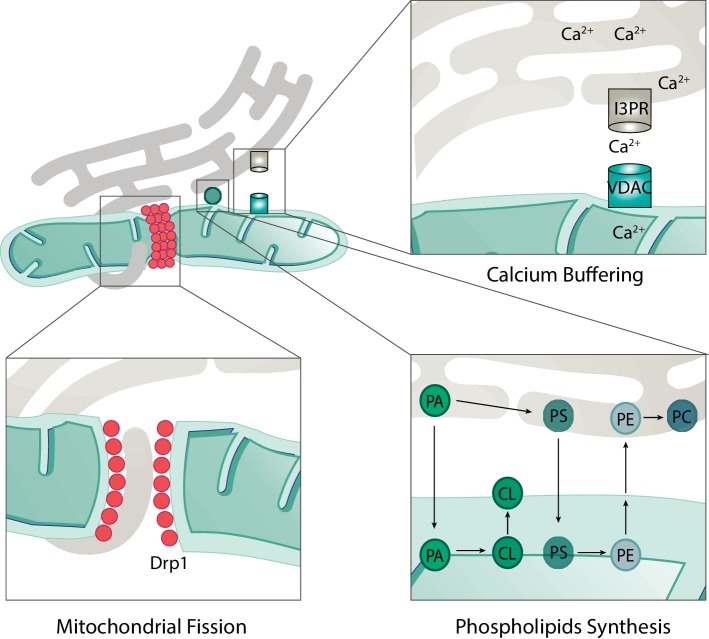
Roles of mitochondria-ER contacts. The physical contacts established between mitochondria and ER are responsible for several cellular processes such as mitochondrial fission, calcium buffering, and phospholipid synthesis. During mitochondrial fission, ER tubules are found in contact with mitochondria in the future fission sites. Drp1 is recruited to these sites and, together with ER tubules, promotes constriction and fission of mitochondria. Calcium (Ca^2+^) is transported from the ER to mitochondria *via* transporters in each membrane. On the ER membrane, Ca^2+^ is exported *via* the inositol 1,4,5-triphosphate receptor (I3PR) to the voltage-dependent anion-selective channel (VDAC) on the mitochondrial outer membrane. For the synthesis of some phospholipids, the required enzymes are found in the mitochondria and not in the endoplasmic reticulum (ER). This implies the transfer of precursor forms of phospholipids from the ER to mitochondria where they can be modified and then re-transferred to the ER. For example, the production of the mitochondrial phospholipid cardiolipin (CL) depends on precursor lipids present at the ER. Phosphatidic acid (PA) is transferred from the ER, across the IMS to the IMM, where it is enzymatically modified to form CL, which is further transported to the OMM. An example of a bidirectional movement of lipids between the ER and mitochondria is the production of phosphatidylethanolamine (PE) and phosphatidylcholine (PC). Phosphatidylserine (PS) is first produced on the ER membrane and then translocated to the OMM. On the OMM, PS is transferred to the IMM where it is enzymatically modified to form PE. Finally, in order to produce PC, the precursor PE must be transferred back to the ER where specific enzymes modify it into PC.

Mitochondria are responsible for a number of cellular and subcellular processes. Consistently, mitochondria form a dynamic network with several other organelles. For example, the junctions formed with the ER are known as the mitochondria-associated ER membrane (MAM) sites and can cover up to 5% of mitochondria (Wu et al., 2018). These junctions are of extreme importance for several processes such as lipids biosynthesis, mitochondrial dynamics, and Ca^2+^ transfer (Figure 5; Szabadkai et al., 2006; Friedman et al., 2011; Rowland and Voeltz, 2012; Kojima et al., 2016; Wu et al., 2018; Doghman-Bouguerra and Lalli, 2019).

The majority of enzymes necessary for lipid biosynthesis are found on the ER membrane. However, synthesis of phosphatidylethanolamine (PE) and phosphatidylcholine (PC), the two most abundant phospholipids, requires lipid trafficking between mitochondria and ER, due to the localization of the required enzymes (Rowland and Voeltz, 2012; Kojima et al., 2016; Tatsuta and Langer, 2017). PE is produced from phosphatidylserine (PS), which is synthetized on the ER membrane. In turn, the enzyme phosphatidylserine decarboxylase, which is responsible for the majority of PE biosynthesis, locates mostly at the IMM of mitochondria (Tatsuta and Langer, 2017; Friedman et al., 2018). Therefore, PS must be transferred to mitochondria. In turn, to produce PC, PE must be transferred back to the ER, again requiring lipid transfer events. In conclusion, the biosynthesis of both PE and PC demonstrates the importance of ER-mitochondria contact sites in lipid biosynthesis (Figure 5).

Interestingly, the ER was shown to be an active regulator of mitochondrial dynamics. ER tubules that contact with mitochondria were found to correlate with the presence of the mitochondrial fission factor Drp1 (Friedman et al., 2011). In agreement with the idea that ER-mitochondria contacts might regulate mitochondrial division, they correlated with the presence of constricted mitochondria, prior to Drp1 recruitment (Figure 5; Friedman et al., 2011). In addition, MFN2 was suggested to be present at the ER, in MAM sites, directly acting as a tether between these two organelles (De Brito and Scorrano, 2008). However, whether ER-mitochondria juxtaposition is promoted (De Brito and Scorrano, 2008; Naon et al., 2016; Basso et al., 2018; McLelland et al., 2018) or inhibited (Cosson et al., 2012; Filadi et al., 2015; Wang et al., 2015) by MFN2 is controversially discussed, depending perhaps on the cellular growth conditions. Interestingly, a role in ER-mitochondria contacts in inhibiting mitophagy was recently shown (Basso et al., 2018; McLelland et al., 2018).

One of the most well-characterized processes where ER-mitochondria contacts are indispensable is Ca^2+^ buffering (Marchi et al., 2017). Ca^2+^ is transferred from the ER through the inositol 1, 4,5-triphosphate receptor (I3PR) to the voltage-dependent anion-selective channel (VDAC) on the OMM (Figure 5; Rizzuto et al., 1998; Szabadkai et al., 2006). In turn, Ca^2+^ influx to the mitochondrial matrix occurs *via* the mitochondrial calcium uniporter (MCU; Kirichok et al., 2004; Baughman et al., 2012). The transfer of Ca^2+^ to mitochondria is required for several mitochondrial proteins or processes, including the TCA cycle enzymes (Bustos et al., 2017). In addition, mitochondrial division has been shown to be affected by Ca^2+^ levels, in a Drp1-dependent manner (Friedman et al., 2011). In fact, some factors required for mitochondrial division and found in MAM sites are regulated by Ca^2+^ binding, as for example MIRO, a protein mainly required for mitochondrial trafficking (Saotome et al., 2008). Finally, Ca^2+^ transferring at the ER-mitochondria contacts was also shown to activate apoptosis (Doghman-Bouguerra and Lalli, 2019). Ca^2+^ influx to mitochondria is able to open the mitochondrial permeability transition pore, leading to the release of cytochrome c and further apoptosis induction (Scorrano et al., 2003).

### Apoptosis

**Figure 6:**
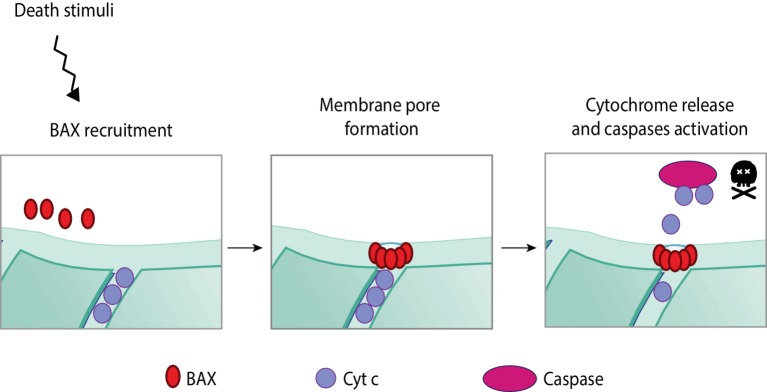
Apoptotic intrinsic pathway mediated by mitochondria. The programmed and regulated cell death, apoptosis, can occur *via* two different pathways—intrinsic or extrinsic—according to the origin of the death stimuli, whether it is intrinsic or extrinsic to the cell. Upon intrinsic death stimuli, such as, for example, DNA damage or oncogene activation, the intrinsic apoptotic pathway is activated, which is mediated by mitochondria. Intrinsic stimuli induce the oligomerization of a pro-apoptotic BcL-2 protein—BAX. These oligomers are able to permeabilize the mitochondrial membrane by pore formation on the OMM. Membrane permeabilization allows the release of pro-apoptotic molecules from the IMS, importantly, cytochrome c. In a complex together with other pro-apoptotic proteins, cytochrome c activates caspases, the effectors of apoptosis.

Apoptosis is a highly regulated programmed form of cell death that occurs in response to stress. The apoptotic cascade can be activated *via* the extrinsic or the intrinsic pathway depending on whether the stress signals are extra or intracellular, respectively (Elmore, 2007; Galluzzi and Vitale, 2018). Both pathways culminate with the activation of caspases, the final effectors of apoptosis. The extrinsic pathway is initiated with the binding of an extracellular death ligand to a cell-surface death receptor. In turn, internal death stimuli are, for example, DNA damage, oncogene activation, the absence of certain growth factors/hormones, or viral infection. The apoptotic intrinsic pathway is mediated by mitochondria (and therefore also known as mitochondrial pathway). It occurs through the release of pro-apoptotic molecules from the IMS to the cytosol, for example, cytochrome c or SMAC/DIABLO (Figure 6; Xiong et al., 2014; Ugarte-Uribe and García-Sáez, 2017).

The major players in the apoptotic mitochondrial pathway are proteins belonging to the Bcl-2 family, which can be divided into pro-survival, pro-apoptotic, or apoptosis initiators (Xiong et al., 2014). Within the pro-apoptotic Bcl-2 proteins, BAX and BAK are the two main regulators (Wei et al., 2001; Ugarte-Uribe and García-Sáez, 2017). BAX is a cytosolic protein that translocates to mitochondria upon apoptotic stimuli, where it oligomerizes (Antonsson et al., 2001). Simultaneously, BAK, which locates to mitochondria, undergoes conformational changes and oligomerization upon death stimuli (Griffiths et al., 1999). Although not completely understood how, both BAX and BAK form pores on the OMM, enabling the release of pro-apoptotic molecules from the IMS to the cytosol (Wang and Youle, 2009). Once in the cytosol, cytochrome c binds to the apoptotic protease activating factor 1 (Apaf-1) (Liu et al., 1996), forming the apoptosome. This complex cleaves and activates the pro-caspase 9, followed by the activation of effector caspases (Xiong et al., 2014).

Interestingly, BAX and BAK can interact with mitofusins and Drp1, thus placing apoptosis in close relation with mitochondrial dynamics (Karbowski et al., 2002; Brooks et al., 2007). However, the impact of mitochondrial dynamics and morphology on apoptosis is still controversially discussed (Xie et al., 2018). On one hand, mitochondrial fragmentation was suggested to induce cell death, because fragmented or clustered mitochondria correlated with increased apoptosis, whereas Drp1 loss-of-function prevented apoptosis (Frank et al., 2001; Huang et al., 2007). BAX was shown to translocate to specific sites on mitochondria during the early stages of apoptosis, which subsequently become mitochondrial fission sites (Karbowski et al., 2002), Consistently, Drp1 was able to permeabilize the OMM by BAX recruitment to mitochondria (Montessuit et al., 2010). Moreover, a pro-apoptotic role of Drp1 by stabilizing ER-mitochondria contact sites was recently shown (Prudent et al., 2015; Prudent and Mcbride, 2017). On the other hand, caspase-3 activation and enhanced apoptosis could be observed in Drp1-deficient mice or derived colon cancer cells, attributing an anti-apoptotic role to mitochondrial fragmentation (Wakabayashi et al., 2009; Inoue-yamauchi and Oda, 2012). Reciprocally, a role of BAK and BAX in the regulation of mitochondrial fusion was proposed (Brooks et al., 2007; Hoppins et al., 2011). First, a role of BAK in promoting mitochondrial fragmentation during apoptosis was suggested, along with the disassociation from MFN2 and association with MFN1 (Brooks et al., 2007). Second, under non-apoptotic conditions, soluble BAK activated mitochondrial fusion *via* MFN2 (Hoppins et al., 2011). In conclusion, the reciprocal relation between mitochondrial dynamics and apoptosis is complex and context-specific.

### Mitophagy

**Figure 7:**
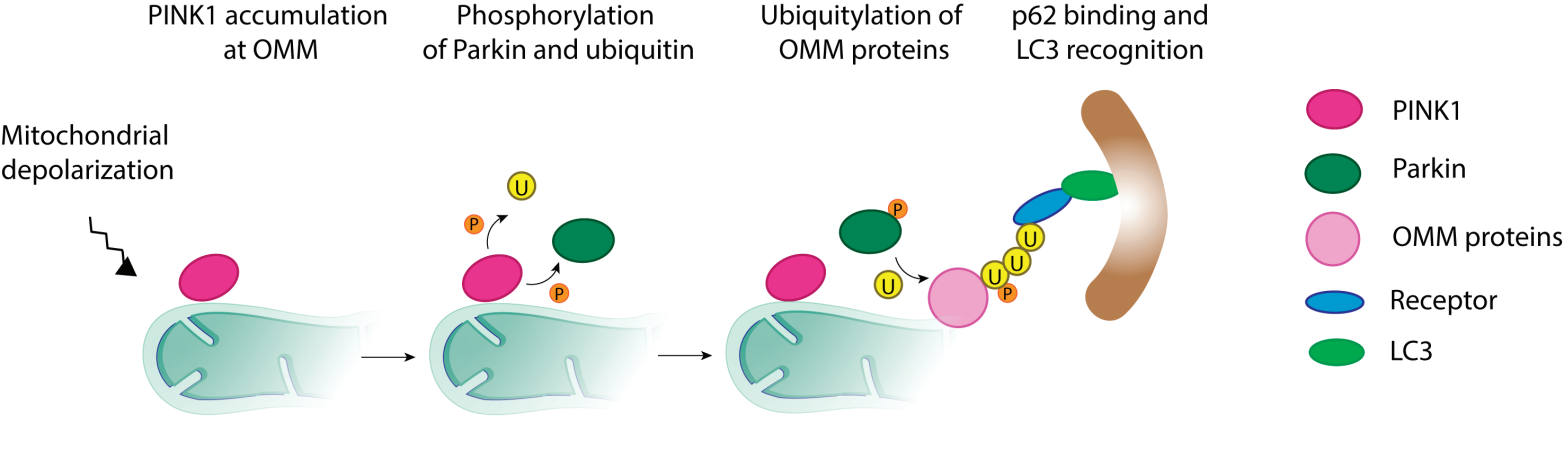
Mitochondrial clearance *via* the ubiquitin-mediated pathway. Upon mitochondrial depolarization, a cascade of events is initiated, which targets damaged mitochondria, or portions of it, for degradation by the autophagy machinery. In a first step, the kinase PINK1 accumulates at the mitochondrial outer membrane and initiates phosphorylation (P) and recruitment of the E3 ligase Parkin. Activated Parkin ubiquitylates (U) several outer mitochondrial membrane proteins such as Mitofusins 1 and 2. Additional phosphorylation of ubiquitin generates a positive feedback loop increasing Parkin recruitment and further ubiquitylation. The formation of ubiquitin chains on mitochondrial surface proteins promotes its binding to lipidated LC3, an autophagosome receptor, *via* the mitochondrial receptors (R) such as optineurin, NDP52, or p62. From this point, the mitochondria, or its fragments, meant to be degraded are surrounded by the autophagosome, which finally fuses with the lysosome for final destruction.

Mitochondria are kept in vigilance by a multi-layered quality control system that protects it against all sorts of stress, ensuring maintenance of healthy mitochondria (Harper et al., 2018; Pickles et al., 2018). Upon extreme stress, such as loss of membrane potential, failure of mitochondrial channels, or severe mitochondrial dysfunction, the quality control mechanism activated is mitophagy. Mitophagy is a selective form of macroautophagy that eliminates damaged mitochondrial proteins, or portions of damaged mitochondrial network. It occurs *via* their engulfment by autophagosomes, which subsequently fuse with the lysosome, where degradation occurs (Figure 7).

Mitophagy requires the presence of specific receptors linking the autophagosome membrane to the mitochondrial portion destined for degradation, and it can be either dependent or independent on ubiquitin. Moreover, in most cases, mitophagy is also dependent on the ubiquitin-like modifier Atg8 (yeast)/LC3 (mammals), whose lipidated and active form (LC3-II) integrates in the autophagosome membrane. However, LC3-independent mitophagy has also been reported (Nishida et al., 2009; Saito et al., 2019). In *Saccharomyces cerevisiae*, mitochondria are targeted *via* the OMM protein receptor Atg32, its binding to Atg8, and, consequently, the activation of mitophagy (Kanki et al., 2009; Okamoto et al., 2009). In mammals, the homologue of Atg32, Bcl2-L-13, was reported to bind LC3-II on the autophagosome membrane and to be required for mitophagy induction (Otsu et al., 2015). Furthermore, other OMM proteins containing an LC3 interacting (LIR) motif, such as BNIP3, NIX and FUNDC1, were also described to target mitochondria for mitophagic destruction (Novak et al., 2010; Rikka et al., 2011; Liu et al., 2012; Wu et al., 2014).

The mostly described factors mediating ubiquitin-dependent regulation of mitophagy are the kinase PINK1 and the E3 ligase Parkin (Figure 7). Upon loss of membrane potential, PINK1 accumulates at the OMM and recruits Parkin to the mitochondria. Once at the OMM, Parkin is phosphorylated by PINK1 and thereby activated (Shiba-Fukushima et al., 2012). Activated Parkin initiates ubiquitylation of several OMM proteins, including MFN1 and MFN2, which immediately leads to loss of fusion events and to mitochondrial fragmentation, characteristic of mitophagy (Gegg et al., 2010; Poole et al., 2010; Tanaka et al., 2010; Ziviani et al., 2010). Furthermore, the poly-ubiquitin chains on surface proteins get bound to LC3-II *via* several adaptors, such as optineurin, NDP52, and p62 (Geisler et al., 2010; Narendra et al., 2010b; Lazarou et al., 2015; Khaminets et al., 2016; Mcwilliams and Muqit, 2017), thus allowing the association of mitochondria to the autophagosomes (Geisler et al., 2010).

In addition to Parkin, the E3 ligase Gp78 also activates mitophagy upon mitochondrial depolarization (Fu et al., 2013). Moreover, other ligases were reported to induce mitophagy as a response to other stress factors, for example, MARCH5, upon disruption of oxygen homeostasis (Daskalaki et al., 2018; Ferrucci et al., 2018; Shefa et al., 2019). In fact, deficiency of O_2_ causes hypoxic stress, but excess of O_2_ may lead to excessive reactive oxygen species, both with toxic consequences for the cells. Consequently, eukaryotes have developed complex systems to maintain their oxygen homeostasis. Not surprisingly, hypoxic stress was shown to induce mitophagy, dependent on the receptor FUNDC1 (Liu et al., 2012; Lampert et al., 2019) and its ubiquitin-dependent regulation by MARCH5 (Chen et al., 2017).

## E3 Ligases Acting on Mitofusins

**Figure 8:**
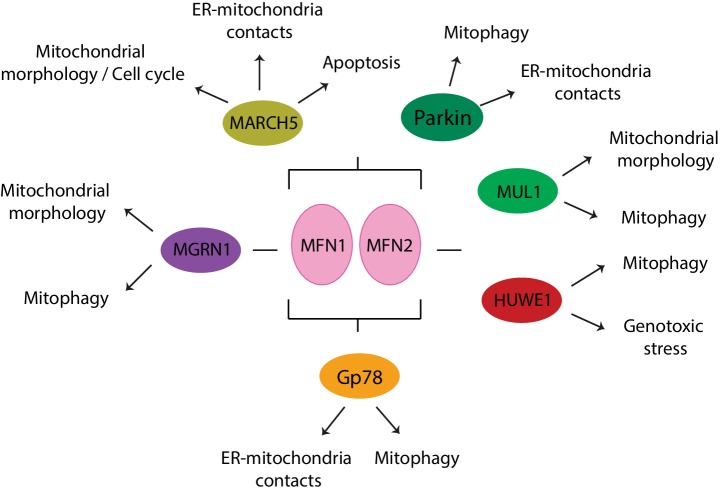
E3 ligases that modify mitofusins and cellular processes associated. MARCH5, Parkin, and Gp78 regulate both mitofusins, whereas MGRN1 affects MFN1 and HUWE1 and MUL1 affect MFN2.

**Figure 9:**
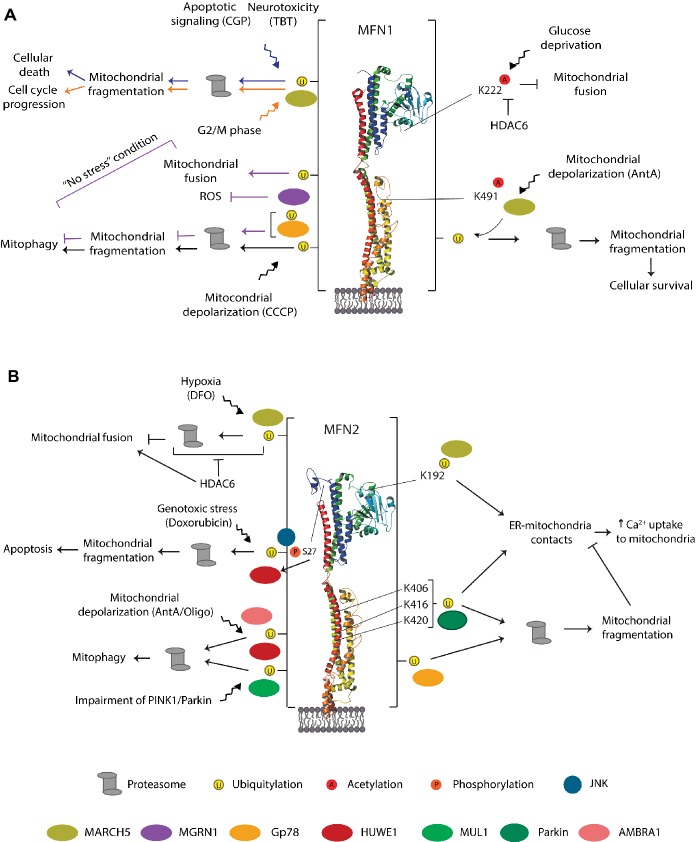
Residues, E3 ligases, and processes regulating MFN1 **(A)** MFN2 **(B)**. Representation of the triggers identified to modify mammalian mitofusins by ubiquitylation, phosphorylation, and acetylation. The enzymes evolved in each case, and the cellular outcome is also depicted. The vertical bar on each side of the structure denotes that the residues modified by ubiquitylation in MFN1 or MFN2 are not known. See text for details.

Various E3 ligases, soluble or membrane embedded and either located to the cytoplasm, the OMM, or the ER, have been shown to regulate either one or both mitofusins, as a response to various physiological or stress-induced conditions (Figures 8, 9A,B). The OMM E3 MARCH5 was implicated in the regulation of mitochondrial morphology, apoptosis, and ER-mitochondria contacts and in responses to toxic stress, *via* both MFN1 and MFN2. In turn, ubiquitylation of MFN2 by the OMM E3 MUL1 is linked to mitochondrial morphology, mitophagy, and neurodegeneration (Cilenti et al., 2014; Yun et al., 2014; Tang et al., 2015). The ER-located E3 Gp78 affected mitophagy and ER-mitochondria contacts, *via* ubiquitylation of both mitofusins (Fu et al., 2013; Wang et al., 2015). Interestingly, the cytosolic E3 MGRN1 was proposed to coordinate the balance between mitochondrial fusion and mitophagy, *via* Gp78 (Mukherjee and Chakrabarti, 2016a,b). Constitutively, MGRN1 promotes a stabilizing ubiquitylation on MFN1, concomitant with a destabilizing ubiquitylation on Gp78, thus preventing mitophagy and instead promoting fusion. By contrast, stress prevented MGRN1-dependent ubiquitylation and turnover of Gp78, consequently leading to MFN1 turnover, mitochondrial fragmentation, and induction of mitophagy. Ubiquitylation of mitofusins by another cytosolic E3, HUWE1, is linked to both genotoxic stress and mitophagy (Leboucher et al., 2012; Di Rita et al., 2018). Finally, the cytosolic E3 Parkin was shown to be recruited to mitochondria under stress, thus ubiquitylating mitofusins and promoting mitophagy, but was also suggested to regulate ER-mitochondria contact sites, both in mammals and in *Drosophila* (Narendra et al., 2008; Gegg et al., 2010; Poole et al., 2010; Tanaka et al., 2010; Ziviani et al., 2010; Glauser et al., 2011; Rakovic et al., 2011).

### MARCH5

The E3 ligase Human membrane-associated RING-CH-V (MARCH5, also named MARCHV or MITOL) is an integral OMM protein with four membrane-spanning segments and a RING-finger domain at its N-terminus (Nakamura et al., 2006; Yonashiro et al., 2006). MARCH5 is associated with the ubiquitylation and degradation of proteins regulating mitochondrial dynamics. It was shown that its overexpression increased mitochondrial tubulation and that its depletion or the presence of a RING-inactive mutant leads to mitochondrial fragmentation (Nakamura et al., 2006; Yonashiro et al., 2006). These results supporting a “pro-tubulation” role of MARCH5, meaning either promoting fusion or *via* inhibition of mitochondrial fission, the later suggested by Xu et al. (2016). However and by contrast, downregulation or RING-inactive mutants of MARCH5 were also shown to induce abnormal elongation of mitochondria (Karbowski et al., 2007; Park et al., 2010). In fact, depending on the circumstances, MARCH5 ubiquitylates or interacts with both fission and fusion components, suggesting a plastic role in the regulation of mitochondrial morphology, as a response to different stimuli.

Consistent with a plastic role of MARCH5, this ligase was reported to control a fine balance of MFN1 levels and mitochondrial fusion, in order to avoid cellular senescence (Park et al., 2014). First, MARCH5 downregulation led to intense elongation of mitochondria, a pro-survival effect (Park et al., 2010). However, persistent downregulation caused aggregation of mitochondria, progressive cellular enlargement and flattening as well as increased senescence (Park et al., 2010). Consistently, the same authors showed that upon mitochondrial stress, caused by Antimycin A (an inhibitor of complex 3 of the respiratory chain), mitochondria first elongate along with increased levels of MFN1. However, excessive MFN1 leads to mitochondrial aggregation and cellular senescence, which is counteracted by MARCH5. MARCH5 interacts with MFN1 (Park et al., 2010), preferentially binding to acetylated MFN1 on K491 (Park et al., 2014), which is conserved in yeast but not in MFN2. Then, MARCH5 assembles K-48-linked ubiquitin chains on MFN1, addressing it for proteasomal degradation (Park et al., 2014). Moreover, cellular senescence of MARCH5 depleted cells could be rescued by further knockdown of MFN1, especially under Antimycin A induced stress (Park et al., 2014). This suggests an important pro-survival role of MARCH5 upon mitochondrial stress *via* MFN1, concomitant with increased acetylation of MFN1, rendering it a preferential substrate for MARCH5-dependent degradation (Park et al., 2014).

A link of MARCH5 with cell death, mediated by ubiquitylation of MFN1, was observed upon the addition of CGP37157 (CGP), an inhibitor of mitochondrial calcium efflux, thus an enhancer of apoptosis (Choudhary et al., 2014). Induction of cell death in prostate cancer cells with CGP led to ubiquitylation and degradation of MFN1. MFN1 turnover was dependent on MARCH5, suggesting that it could directly modify MFN1. Moreover, MFN1 depletion in prostate cancer cells increased the cell death response to CGP. Therefore, a pro-apoptotic role of MARCH5 and the potential therapeutic benefits of MFN1 inhibition are suggested (Choudhary et al., 2014). Consistently, in induced pluripotent stem cells (iPSCs), a decrease in cell viability and ATP content, as well as mitochondrial fragmentation, was observed with tributyltin (TBT; Yamada et al., 2016), an endocrine disruptor that causes neurotoxicity and immunotoxicity (Kotake, 2012). TBT led to a decrease in MFN1 levels, which depended on MARCH5, presumably *via* direct ubiquitylation of MFN1 (Yamada et al., 2016).

The apparent contradictory observations of a pro- and anti-survival role of MARCH5 upon Antimycin A or CGP/TBT treatment, respectively, could be explained by the extension of MFN1 overexpression achieved by each stress. Consistently, the levels of acetylated MFN1 probably regulate a fine-tuned balance of fusion activity as well. Indeed, the ubiquitin binding deacetylase HDAC6, mostly cytosolic, was shown to interact with MFN1, mainly under glucose deprivation (Lee et al., 2014). Interestingly, HDAC6 also bound to MFN2, but its acetylation or interaction with HDAC6 was not altered under glucose deprivation (Lee et al., 2014). Importantly, acetylation of MFN1 at K222 was shown to inhibit its fusogenic activity. Moreover, HDAC6-dependent deacetylation of MFN1 ameliorated ROS production during glucose starvation, supporting a role of MFN1-mediated fusion to cope with metabolic stress (Lee et al., 2014). Therefore, HDAC6 promotes MFN1-dependent hyperfusion, observed both upon glucose starvation in cells and upon fasting in mice (Lee et al., 2014). In conclusion, various stress conditions lead to MARCH5-dependent ubiquitylation and degradation of MFN1.

In addition to MFN1, MARCH5 and HDAC6 regulate stress-induced MFN2 turnover (Kim et al., 2015). MARCH5 interacts with MFN2 (Nakamura et al., 2006), which occurs between the C-terminal domain of MARCH5 and the HR1 domain of MFN2 (Sugiura et al., 2013). Moreover, MARCH5 was shown to be responsible for ubiquitylation and degradation of MFN2 under hypoxic stress, provoked by adding deferoxamine (DFO), in cells lacking HDAC6 (Kim et al., 2015). However, HDAC6 bound strongly to MFN2 in the presence of DFO, thus inhibiting MFN2 turnover and preventing mitochondrial fragmentation (Kim et al., 2015). Therefore, HDAC6 can protect mitochondria and activate adaptive mitochondrial fusion under hypoxic stress (*via* deacetylation of MFN2) and under starvation (*via* deacetylation of MFN1). In both cases, deacetylation of mitofusins induced their fusogenic capacity by preventing ubiquitylation and degradation by MARCH5.

The regulation of MFN1 and MFN2 by MARCH5 was also observed in the absence of external stress, being instead either linked to cell cycle, *via* MFN1, or linked to ER-mitochondria exchanges, *via* MFN2. Cell cycle transitions are accompanied by alterations in mitochondrial morphology, which are actively regulated by both fusion and fission proteins (Mitra, 2013; Horbay and Bilyy, 2016). During the G2/M phase, mitochondria fragment; then, after cellular division, they fuse again (Mitra, 2013). Consistent with the fragmentation, it was shown that MARCH5 ubiquitylates MFN1 at G2/M, addressing it for proteasomal degradation (Park and Cho, 2012). By contrast, a non-proteolytic role of MARCH5 acting on MFN2 was demonstrated, through the addition of K63-linked polyubiquitin chains at K192, located on the GTPase domain and not conserved in MFN1 (Sugiura et al., 2013). Despite the suggestion that MFN2 also locates to the ER, MARCH5 only ubiquitylated MFN2 present at the mitochondria, not ER-associated MFN2. This created a non-proteolytic tag, instead promoting the formation of MFN2 higher oligomers, important to maintain ER-mitochondria contacts (Sugiura et al., 2013). Consistently, MARCH5 knockdown caused MFN2 decrease at MAM sites as well as reduced co-localization of mitochondria and ER, which was rescued upon re-expression of MARCH5. Moreover, a decrease in mitochondrial Ca^2+^ uptake could be observed, suggesting a functional role of MFN2-mediated contact sites, dependent on MARCH5 ubiquitylation of MFN2 (Sugiura et al., 2013).

### HUWE1

HUWE1 is a multifunctional E3 ligase belonging to the HECT-domain E3 ligase family, therefore forming a ubiquitin-thioester intermediate with ubiquitin, before transferring it to the substrate (Bernassola et al., 2008). HUWE1 is composed by an ubiquitin-associated domain, a WWE domain (involved in proteolysis), a BH3 domain (common to the family), and two N-terminal domains. Finally, the HECT domain is found in its C-terminus (Kao et al., 2018). HUWE1 mediates not only K48- and K63-linked poly-ubiquitylation (Adhikary et al., 2005; Zhao et al., 2008) but also mono-ubiquitylation (Parsons et al., 2009) and K11-/K6-linked ubiquitylation (Michel et al., 2017), therefore being suggested to assemble a powerful ubiquitin combination for proteasomal turnover (Meyer and Rape, 2014). This E3 ligase is majorly known for regulation of proliferation, differentiation, and apoptosis, being therefore associated with cancer and metastasis (Kao et al., 2018).

MFN2 was recently shown to be modified in a HUWE1-dependent manner with K6-linked polyubiquitin chains (Michel et al., 2017). Moreover, it was demonstrated that HUWE1 ubiquitylates MFN2 in response to genotoxic stress (Leboucher et al., 2012) and upon induction of mitophagy (Di Rita et al., 2018), thereby addressing MFN2 for proteasomal degradation. First, in sarcoma cells, HUWE1 ubiquitylated MFN2 upon activation of the c-Jun N-terminal kinase (JNK) pathway, by the addition of Doxorubicin, a genotoxic stressor well known for inducing apoptosis (Leboucher et al., 2012). Doxorubicin led to mitochondrial fragmentation and to MFN2 ubiquitylation and phosphorylation at serine 27, signaling proteasomal-dependent loss of MFN2. The authors identified the JNK as the stress-activated kinase phosphorylating MFN2. In addition, MFN2 bound to the BH3 domain of HUWE1, suggesting a role in apoptosis. Consistently, knockdown of HUWE1 prevented apoptosis, which was restored upon further knockdown of MFN2. In summary, this study proposes that phosphorylation of MFN2 by JNK leads to recruitment of the E3 ligase HUWE1 to phosphorylated MFN2 and subsequent acceleration of its degradation by the proteasome. Consequently, degradation of MFN2 leads to enhanced mitochondrial fragmentation and apoptosis (Leboucher et al., 2012).

Second, a role of HUWE1 in mitophagy induction was recently identified. This was observed upon the addition of Oligomycin (an inhibitor of complex 5 of OXPHOS) and Antimycin A, by a pathway mediated by AMBRA1 (Di Rita et al., 2018). AMBRA1 is an inducer of autophagy and a regulator of mitophagy, involved in both PINK1/Parkin-dependent and independent pathways, through binding to LC3 (Strappazzon et al., 2015). HUWE1 interacted with AMBRA1, especially under mitophagy conditions, and translocated to mitochondria. Moreover, both HUWE1-dependent ubiquitylation and proteasomal-dependent degradation of MFN2 were observed. Importantly, HUWE1 depletion impaired AMBRA1-mediated mitophagy (Di Rita et al., 2018).

### Gp78

Glycoprotein 78 (Gp78) is an ER membrane-anchored E3 ubiquitin ligase (Nabi and Raz, 1987). Gp78 is inhibited by the ubiquitous cytokine autocrine motility factor (AMF) and was first identified as the autocrine motility factor receptor (AMFR), for its role in a signaling cascade regulating cancer cell motility and metastasis (Nabi et al., 1990; Silletti et al., 1991). Gp78 regulates protein quality control *via* the ER-associated degradation machinery (ERAD; Fang et al., 2001). ERAD is responsible for the degradation of misfolded or functionally denatured proteins from the ER, occurring *via* proteasomal degradation after their retro-translocation to the cytosol (Mehrtash and Hochstrasser, 2018). This E3 ligase possesses a G2BR domain, for E2-binding, has five N-terminal transmembrane domains, and, in its C-terminus, has the RING-Finger and VIM domain facing the cytosol (Joshi et al., 2017). The E3 ligase activity of Gp78 is associated with cell signaling and motility, metabolism, neurodegeneration, and cancer/metastasis (Liottaab et al., 1986; Watanabes et al., 1991; Nabi et al., 1992; Luo et al., 2002).

Regarding mitofusins, a role of Gp78 was observed for mitophagy (Fu et al., 2013) and ER-mitochondria contact sites (Wang et al., 2015). First, overexpression of Gp78 was shown to induce mitochondrial fragmentation, dependent on its E3 ligase activity (Fu et al., 2013). Moreover, mitochondrial loss was observed, further aggravated upon mitochondrial uncoupling with CCCP, but rescued by Gp78 downregulation, pointing to its role in mediating mitophagy. Consistently, Gp78 expression was shown to partially recruit LC3 to the ER, co-localizing with Gp78 itself (Fu et al., 2013). Moreover, recruitment of LC3 to Gp78-positive ER domains was dramatically increased upon CCCP treatment, supporting a direct role of Gp78 for clearance of damaged mitochondria. Concomitantly, decreased levels of MFN1 and MFN2 were observed, especially in the presence of CCCP, an effect prevented by proteasomal inhibition (Fu et al., 2013). Moreover, Gp78 interacted with both mitofusins (Fu et al., 2013) and its inhibitor, AMF, prevented Gp78-induced degradation of both MFN1 and MFN2 (Shankar et al., 2013). However, knockdown of MFN1, but not of MFN2, inhibited induction of mitophagy, suggesting that albeit degraded, MFN1 is required for mitophagy. In summary, the role of Gp78 in mitophagy appears to be specifically dependent on MFN1.

In addition to mitophagy, Gp78 promoted contact sites between the ER and mitochondria, which were functional in calcium transfer (Wang et al., 2015). However, despite Gp78 being mostly localized to smooth-ER (Benlimame et al., 1998), it specifically affected contacts of mitochondria to rough ER, in fibrosarcoma cancer cells (Wang et al., 2015). Moreover, in this case, the selective regulation of rough ER by Gp78 depended specifically on the presence of MFN2. Given that MFN2 levels increased upon downregulation of Gp78, the role of the ligase in promoting rough ER-mitochondria contacts might occur *via* ubiquitylation and subsequent degradation of MFN2 (Wang et al., 2015). By contrast, although the levels of MFN1 also increased in the absence of Gp78, MFN1 knockdown did not affect rough ER-mitochondria contacts. Instead, MFN1 knockdown inhibited the contacts between mitochondria and smooth ER, which were not affected by Gp78 knockdown or by the addition of its inhibitor AMF. In summary, both MFN1 and MFN2 behaved as inhibitors of ER-mitochondria contact formation, albeit through different mechanisms and at different ER sites (Wang et al., 2015).

### MGRN1

The E3 ligase Mahogunin Ring Finger-1 (MGRN1) was first discovered to be the gene mutated in a color-coat mutant mice, *mahoganoid* (Phan et al., 2002; He et al., 2003; Upadhyay et al., 2016). MGRN1 is a soluble E3 ligase, however, locating to the cytoplasm, plasma membrane, endosomes, and nucleus (Bagher et al., 2006).

Interestingly, a relation between MGRN1 and both Gp78 and MFN1 was suggested, affecting both mitophagy and mitochondrial fusion. First, the levels of Gp78 itself were negatively regulated by MGRN1, an effect observed in the absence of external stress (Mukherjee and Chakrabarti, 2016a,b). Depletion of MGRN1, or deletion of the RING-finger domain, caused perinuclear clustering of mitochondria and increased oxidatively modified proteins, indicative of ROS (Mukherjee and Chakrabarti, 2016a,b). Moreover, MGRN1-dependent ubiquitylation of Gp78 addressed it for proteasomal degradation. Thus, by repressing Gp78, MGRN1 indirectly prevented mitophagy, MFN1 turnover, and perinuclear clustering. However, depolarization conditions compromised ubiquitylation of Gp78 by MGRN1, thus rescuing the levels of Gp78 and favoring mitophagy of damaged mitochondria (Mukherjee and Chakrabarti, 2016b). Indeed, cells expressing the Ring-Finger mutant MGRN1 reveal higher propensity for mitophagy: they displayed higher levels of LC3-positive mitochondria and presented increased co-localization of mitochondria with p62-positive autophagic vesicles.

Second, MGRN1 also preserved MFN1 stability by directly interacting with it. Moreover, expression of a RING-finger mutant impaired higher oligomerization of MFN1 and mitochondrial fusion (Mukherjee and Chakrabarti, 2016a). Thus, MGRN1 was suggested to actively promote mitochondrial fusion *via* non-degradative ubiquitylation of MFN1, consistent with previous observations (Yue et al., 2014). This is reminiscent of the E3 ligase SCF^Mdm30^ that modifies the yeast mitofusin Fzo1 (Fritz et al., 2003; Escobar-henriques et al., 2006; Cohen et al., 2008, 2011; Anton et al., 2011) with a stabilizing ubiquitylation (Anton et al., 2013; Simões et al., 2018). Indeed, a dual and interdependent balance between constitutive/non-degradative ubiquitylation of MFN1 *vs.* stress-induced/degradative ubiquitylation resembles the regulation of Fzo1 in yeast (Anton et al., 2013; Simões et al., 2018).

### Parkin

Parkin, an E3 ligase associated with Parkinson’s disease (Kitada et al., 1998), belongs to the RING-between-RING family of E3 ligases (Smit and Sixma, 2014; Walden and Rittinger, 2018): it has an N-terminal Ub-like (UBL) domain, a zinc-binding domain (RING0, unique to Parkin), a RING domain (RING1, a canonical domain), and two linear zinc-binding folds (IBR and RING2; Panicker et al., 2017). Importantly, Parkin recruitment to the mitochondria requires PINK1 (Geisler et al., 2010; Matsuda et al., 2010; Vives-Bauza et al., 2010; Narendra et al., 2010a), namely its kinase activity and localization at the mitochondrial surface (Okatsu et al., 2012; Shiba-Fukushima et al., 2012). Moreover, crystal structures revealed that Parkin assumes an auto-inhibited conformation that is activated by undergoing major structural rearrangements, which require PINK1-dependent phosphorylation at serine 65 of its UBL domain. In addition, it requires binding of ubiquitin itself, also phosphorylated by PINK1 at serine 65 (Riley et al., 2013; Spratt et al., 2013; Wauer and Komander, 2013; Caulfield et al., 2014; Kane et al., 2014; Kazlauskaite et al., 2014; Koyano et al., 2014; Swatek and Komander, 2016; Wauer et al., 2016; Kumar et al., 2017; Mcwilliams et al., 2018; Gladkova et al., 2019). Parkin is able to ubiquitylate itself as well as a large variety of both cytosolic and OMM proteins. Mono-ubiquitylation and K63-, K48-, K11-, and K6-linked poly-ubiquitylation have been reported for this E3 ligase (Cunningham et al., 2015; Seirafi et al., 2015; Mouton-liger et al., 2017). Despite its diverse functions (Scarffe et al., 2014), the extensive body of literature regarding Parkin and its E3 ligase activity is mainly gathered from its role on mitochondrial clearance (Harper et al., 2018; Pickles et al., 2018), whereby mitofusins are ubiquitylated. Nevertheless, Parkin has also been shown to act on mitofusins for the regulation of ER-mitochondria contact sites (Basso et al., 2018; McLelland et al., 2018).

Among the E3 ligases acting on mitofusins, Parkin is definitely the most well studied. Parkin was initially shown to translocate from the cytoplasm to depolarized mitochondria in mammalian cells (Narendra et al., 2008). This study led to the development of the hypothesis that Parkin not only is responsible for the ubiquitylation of proteins, leading to their subsequent degradation by the UPS, but also acts on the selective elimination of impaired mitochondria. Strikingly, this dramatically raised the interest of many researchers. Shortly after, several mitochondrial ubiquitylation targets of Parkin were identified (Chan et al., 2011). These included dMfn (MARF) in *Drosophila* (Poole et al., 2010; Ziviani et al., 2010) and MFN1 and MFN2 in murine and mammalian cells (Gegg et al., 2010; Tanaka et al., 2010; Glauser et al., 2011; Rakovic et al., 2011), as well as other OMM proteins like VDAC1, Fis1, or Tom20 (Chan et al., 2011). The ubiquitylation of several mitochondrial proteins by Parkin precedes mitophagy and is accomplished by the proteasome, independently of autophagy (Chan et al., 2011; Sarraf et al., 2013). Furthermore, this was shown to be essential for mitophagy, since inhibition of the 26S proteasome fully abrogated Parkin-mediated mitophagy (Tanaka et al., 2010; Chan et al., 2011). In conclusion, upon mitochondrial depolarization, Parkin mediates ubiquitylation of both mitofusins, thus addressing them for proteasomal turnover. However, whether mitofusins are actively required for Parkin-mediated mitophagy is still controversial (Narendra et al., 2008; Chan et al., 2011; Chen and Dorn, 2013), as discussed later.

Interestingly, a direct role of MFN2 in preventing mitophagy dependent on PINK1/Parkin, by keeping mitochondria tethered to the ER, was recently demonstrated (Basso et al., 2018; McLelland et al., 2018). First, depolarization impaired the connection between mitochondria and the ER, a process that depended on proteasomal turnover and was further exacerbated by overexpression of Parkin (McLelland et al., 2018). In fact, turnover of both mitofusins is a very early event after the addition of CCCP (Chan et al., 2011; Sarraf et al., 2013; McLelland et al., 2018). MFN2 was phospho-ubiquitylated, as expected, and its subsequent degradation contributed to the recruitment of Parkin to mitochondria, thus activating the general ubiquitylation of other outer membrane proteins and accelerating mitophagy (McLelland et al., 2018). Consistently, the Parkin-resistant MFN2 HR1 mutants K406R, K416R and K420R failed to induce mitophagy, a specific effect since their capacity to promote mitochondrial fusion was not affected.

Second, in contrast, Parkin-mediated ubiquitylation of mitofusins, also at K416, promoted ER-mitochondria contact sites (Basso et al., 2018). Indeed, downregulation of Parkin in *Drosophila* or mouse embryonic fibroblast (MEF) cells led to decreased ubiquitylation of mitofusins, concomitant with a significant decrease in ER-mitochondria contacts. Moreover, the ubiquitin dead mutant of dMFN, corresponding to K416R in mammals, was also deficient in establishing ER-mitochondria contacts and in mitochondrial Ca^2+^ uptake (Basso et al., 2018). These results suggest that ubiquitylation of dMfn at “lysine 416” regulates physical and functional ER-mitochondria contacts in *Drosophila*. Finally, expressing an ER-mitochondria synthetic linker rescued locomotor deficits associated with Parkinson’s disease. In conclusion, although somehow contradictory, both studies suggest an active role of MFN2 in preventing mitophagy by tethering mitochondria to the ER. It is possible that in the absence of external stress mild MFN2 ubiquitylation by Parkin keeps the two organelles together. Then, upon depolarization, excessive ubiquitylation occurs instead targeting MFN2 for proteasomal turnover, again reminiscent of findings in yeast (Anton et al., 2013; Simões et al., 2018).

### MUL1/MULAN/MAPL/GIDE

Like MARCH5, the E3 ligase MUL1 is an integral outer membrane protein regulating mitochondrial dynamics. MUL1/MULAN was first identified as an NF-κB activator with E3 ligase activity (Matsuda et al., 2003). Later, this E3 ligase was identified as a mitochondrial protein, being also known as mitochondria-anchored protein ligase (MAPL) or growth inhibition and death E3 (GIDE) ligase (Li et al., 2008; Neuspiel et al., 2008; Zhang et al., 2008). MUL1 has two transmembrane domains, with its RING-finger domain facing the cytosol, whose overexpression induces fragmentation and perinuclear clustering of mitochondria (Li et al., 2008; Neuspiel et al., 2008). A clear role of MUL1/MAPL as a SUMO E3 ligase was demonstrated (Braschi et al., 2009). For example, MUL1/MAPL acts in the formation of mitochondria-derived vesicles addressed to peroxisomes (Neuspiel et al., 2008; Sugiura et al., 2014), in inflammation (Barry et al., 2018), and in innate immunity (Doiron et al., 2017). In addition, other SUMO-dependent roles were shown, in the regulation of mitochondrial fission (Braschi et al., 2009) and activation of apoptosis, by stabilizing ER-mitochondria contact sites *via* Drp1 SUMOylation (Prudent et al., 2015). Consistently, Zhang et al. (2008) reported MUL1/GIDE as a pro-apoptotic enzyme. However, in what regards the regulation of mitofusins by MUL1, so far only ubiquitin ligase activity was shown (Yun et al., 2014; Tang et al., 2015).

MUL1 was suggested to compensate for PINK1 and Parkin deficiencies, for example, by ubiquitylating mitofusin in *Drosophila*, dMfn/MARF (Yun et al., 2014), and MFN2 in mammals (Tang et al., 2015), thus contributing to mitochondrial integrity by promoting mitophagy. In the fly, overexpression of dMfn aggravated the lethality and neurodegeneration phenotypes seen upon PINK1/Parkin deficiency (Yun et al., 2014). Moreover, these deficiencies could be compensated by MUL1-dependent ubiquitylation and proteasomal degradation of dMfn. Consistently, loss of MUL1 resulted in an increase in dMfn levels. Importantly, increased levels of dMfn, observed in dopaminergic neurons and muscle of PINK1/Parkin mutant flies, could be rescued upon MUL1 overexpression. Moreover, HeLa cells exposed to cycloheximide presented decreased MFN1 and MFN2 levels, which could be stabilized upon MUL1 silencing (Yun et al., 2014). Consistently, in the mouse model for neurodegeneration, mnd2, where MUL1 accumulated, the levels of MFN2 decreased and mitophagy was enhanced (Cilenti et al., 2014). Moreover, a role of MUL1 in the degradation of MFN2 was suggested in the context of dopaminergic (DA) neuronal loss (Tang et al., 2015), closely related to Parkinson’s and Alzheimer’s diseases. Indeed, Parkinson’s-linked mutations of VPS35, a retromer component for endosomal trafficking, correlated with increased ubiquitylation and decreased levels of MFN2, dependent on the proteasome (Tang et al., 2015). In addition, these neurons presented mitochondrial fragmentation, along with impaired OXPHOS, pointing to mitochondrial dysfunction. Finally, MUL1 inhibition re-increased MFN2, suggesting that MUL1 directly ubiquitylates MFN2, signaling its degradation by the proteasome (Tang et al., 2015). Together, these results indicate that MUL1 compensates for Parkinson’s phenotypes, caused by the loss of PINK1/Parkin or VSP35, by decreasing MFN2.

## Mitofusins: Pro- or Anti-Mitophagic Proteins?

**Figure 10:**
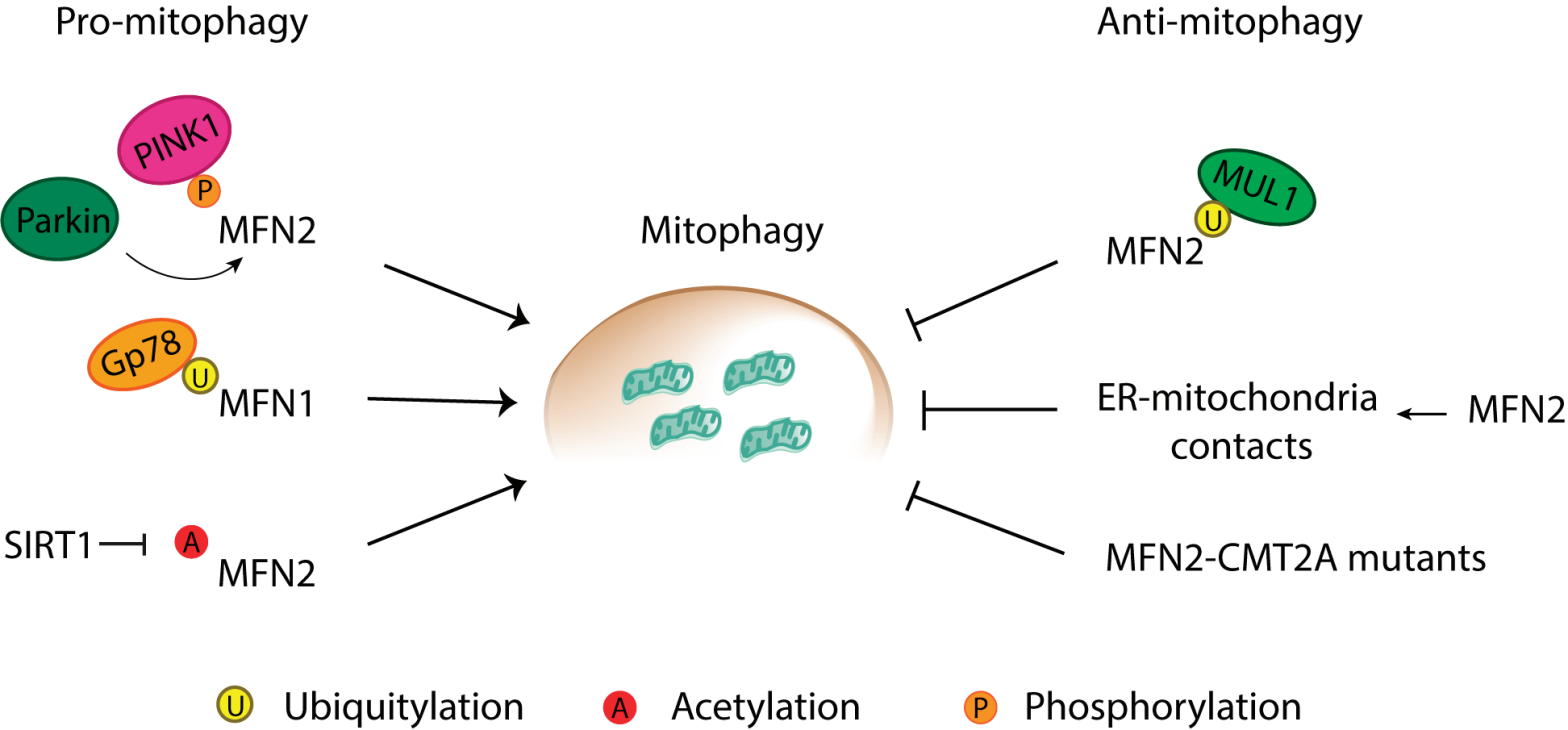
Pro- and anti-mitophagic roles of mitofusins. Mitofusins are reported to induce and inhibit mitophagy through a variety of processes. Pro-mitophagic roles of mitofusins can be promoted by (1) phosphorylation of MFN2 by PINK1, and subsequent recruitment of Parkin to mitochondria, (2) Gp78-mediated ubiquitylation of MFN1, and (3) SIRT1-mediated deacetylation of MFN2. Anti-mitophagic roles have been described for Mitofusin 2 *via* (1) MULAN-mediated ubiquitylation, (2) increase of ER-mitochondria contacts directly mediated by MFN2, and (3) mutations in MFN2 associated with Charcot-Marie-Tooth Type 2A (CMT2A) neuropathy.

During mitophagy induction, mitofusins are clearly among the first substrates to be ubiquitylated by Parkin (Chan et al., 2011; Sarraf et al., 2013; McLelland et al., 2018). However, it was first reported that the absence of both mitofusins did not affect mitophagy (Narendra et al., 2008; Chan et al., 2011). Nevertheless, mutations or modifications of MFN1 and MFN2, either by ubiquitylation, acetylation, or phosphorylation, have also been proposed to directly affect mitophagy (Figure 10). As detailed below, whether mitophagy is enhanced or instead repressed by mitofusins and their ubiquitylation is still controversially discussed.

### Pro-mitophagic Role

An active and essential role of MFN2 for mitophagy was proposed under diverse mitophagy induction conditions and different cell lines, tissues, and animal models. First, MFN2-phosphorylation by PINK1 was required for recruiting Parkin to damaged mitochondria (Chen and Dorn, 2013). This study could show that Parkin binds to MFN2 in a PINK1-dependent manner after which PINK1 phosphorylates MFN2. Phosphorylated MFN2 further promotes ubiquitylation of other OMM proteins by Parkin, targeting mitochondria for degradation. This model was supported by the observation that MFN2 depletion from murine cardiomyocytes prevented Parkin translocation to mitochondria upon membrane depolarization and, consequently, decreased mitophagy levels (Chen and Dorn, 2013). Consistently, depletion of MFN1 and MFN2 caused accumulation of defective mitochondria, but no increase in mitophagy levels was observed (Song et al., 2015). These results suggest that ablation of both mitofusins interferes with a step in the mitophagic process, stopping defective mitochondria from being degraded and leading to their accumulation, pointing, thereby, to the fact that both MFN1 and MFN2 are required for mitochondrial clearance in the murine cardiac system. However, it is important to note that other studies have reported that mitophagy still occurs in the absence of MFN1 or MFN2, pointing to the existence of other proteins that serve as Parkin receptors upon mitophagy induction (Narendra et al., 2008; Chan et al., 2011).

Further supporting a pro-mitophagic role, MFN1-knockdown inhibited mitophagy that was caused by overexpression of Gp78. (Fu et al., 2013). Induction of mitophagy by Gp78 and MFN1 was Parkin-independent (Fu et al., 2013). Moreover, MFN2 was also not required. However, MFN2 was reported as a central player in autophagosome-lysosome formation in human cardiomyocytes (Zhao et al., 2012). Deletion of MFN2 in cardiomyocytes led to the extensive accumulation of autophagosomes, in response to ischemia-reperfusion stress, a condition that induces mitophagy (Zhao et al., 2012). Nevertheless, both autophagosome and lysosome formation remained unaltered (Zhao et al., 2012). Instead, autophagosome accumulation was due to marked retardation of the fusion step between autophagosomes and lysosomes, in the absence of MFN2, a phenotype rescued by re-expression of MFN2 (Zhao et al., 2012).

Additional evidences for a pro-mitophagic role of MFN2 were provided in murine skeletal muscle, in the context of aging (Sebastián et al., 2016). Aging in mice was accompanied by a decrease in MFN2 levels and, consistently, MFN2 depletion generated aging signatures (Sebastián et al., 2016). In parallel, MFN2 ablation impaired autophagy and led to excessive mitochondrial dysfunction (Sebastián et al., 2016). In conclusion, during aging MFN2 levels decreased, consequently impairing mitophagy. Also in the context of aging, MFN2 was reported to induce mitophagy in aged human liver cells upon ischemia/reperfusion (I/R) injury (Chun et al., 2018). This study reported a pro-mitophagic role of MFN2, not promoted by the canonical Parkin-dependent ubiquitylation of MFN2, but by another type of PTM: deacetylation *via* sirtuin 1 (SIRT1). Overexpression of either MFN2 or SIRT1 alone failed to rescue I/R injury, mitochondrial dysfunction, and cell death. However, their co-expression promoted autophagy in aged hepatocytes (Chun et al., 2018). Furthermore, the authors showed that deacetylation of MFN2 by SIRT1 occurs at K655 and K662 residues, located in the C-terminus, and directly linked these modification to an increase in autophagy (Chun et al., 2018).

Finally, supporting pro-mitophagic evidences were shown in primary cultured neurons, where I/R injury phenotypes were ameliorated by MFN2 expression and aggravated by its downregulation (Peng et al., 2018). In fact, MFN2 expression led to increased autophagosome formation and fusion with lysosomes (Peng et al., 2018), suggesting once again an important role of MFN2 factor for mitochondrial clearance.

### Anti-mitophagic Role

In the context of the neuropathy Charcot-Marie-Tooth Type 2A (CMT2A), which is caused by dominant-negative mutations in MFN2, it was suggested that MFN2 behaves as an inhibitor of mitophagy (Rizzo et al., 2016). Indeed, motor neurons derived from iPSCS generated from CMT2A fibroblasts had an increased autophagic flux (Rizzo et al., 2016). This suggests an anti-mitophagic role of mutant MFN2 in CMT2A patients. Consistently, in the CD4^+^ T immune system cells, and in the presence of several stimuli such as rapamycin, ionomycin, or starvation, overexpression of MFN2 led to an impairment in autophagy (Ying et al., 2017).

Moreover, an inhibitory effect of MFN2 toward mitophagy was also suggested in the context of its function as an ER-mitochondria tether. ER-mitochondria contacts are destroyed during mitochondrial clearance process, and, consistently, a reduction in these contacts leads to an increase in mitochondrial degradation (McLelland et al., 2018). Importantly, MFN2 directly promoted contacts between mitochondria and ER, therefore preventing mitophagy (McLelland et al., 2018). Moreover, degradation of MFN2 was necessary for mitophagy to occur. In fact, ubiquitylated MFN2 was suggested to be the active form of MFN2 in promoting ER-mitochondria tethering (Basso et al., 2018). Finally, another anti-mitophagic role of MFN2 was proposed, upon compromised PINK1/Parkin, which depended on ubiquitylation of MFN2 by MUL1, followed by proteasomal degradation (Yun et al., 2014).

## Disease-Associated Roles of MFN2

A broad spectrum of disorders has been linked to mutations or altered levels in MFN2, underlining its physiological relevance (Figure 4). MFN2 correlation with disease also supports the idea that MFN2, more than MFN1, is involved in several other disease-relevant roles besides mitochondrial fusion.

First, MFN2 mutations cause a rare neurodegenerative disease. In addition, a link of MFN2 to the most common neuropathies has also been suggested. Importantly, so far no cure is possible for these diseases, being only symptomatic treatments available. Second, a role of MFN2 in mitophagy or ER-contact sites preventing heart failure was proposed. Finally, MFN2 is also linked to diabetes, an aspect of crucial importance in our present society.

### Neurodegeneration

The major causal link between MFN2 dysfunction and disease lies with the CMT2A neuropathy (Züchner et al., 2004; Verhoeven et al., 2006). CMT2A is a subtype of an incurable neuropathy, Charcot-Marie-Tooth (CMT), characterized by progressive distal weakness, muscular atrophy, and sensory abnormalities, affecting 1 in 2,500 people (Tazir et al., 2013, 2014; El-abassi et al., 2014; Stuppia et al., 2015; Stojkovic, 2016; Barbullushi et al., 2019). CMT is one of the most common inherited neurological diseases, usually inherited as an autosomal dominant trait but sometimes as autosomal recessive and X-linked trait. CMT2A subtype presents an earlier onset, with motor symptoms mainly affecting the lower limbs. More than hundred MFN2 mutations are described as causative of CMT2A, comprising one-fifth of all CMT2A cases (Stojkovic, 2016; Dohrn et al., 2017). Strikingly, MFN2 mutations account for approximately 90% of the most severe cases of CMT (Feely et al., 2011).

To date, the disease-underlying functions of MFN2 have not been identified. Recent studies with CMT2A-associated mutations highlighted the importance of carefully addressing membrane potential, apoptosis, ER-mitochondria contacts, and mitophagy in CMT2A, further suggesting fusion-independent roles of Mitofusin 2 (Saporta et al., 2015; Rizzo et al., 2016; Bernard-Marissal et al., 2018; Larrea et al., 2019). Two very recent studies support a pathogenic role of reduced ER-mitochondria contacts caused by MFN2 mutants associated with CMT2A. First, the presence of the most common CMT2A disease mutant MFN2^R94Q^ in patient-derived fibroblasts, primary neurons, and *in vivo* motor neurons of CMT2A mouse model, impaired ER-mitochondria contacts (Bernard-Marissal et al., 2018). Importantly, ER stress, Ca^2+^ defective uptake, and alteration in the axonal transport of mitochondria, could also be observed. Second, the extent of ER-mitochondria contacts also diminished with three different CMT2A variants: MFN2^R364W^, MFN2^M376V^, and MFN2^W740S^ (Larrea et al., 2019). Moreover, phospholipid synthesis and trafficking were affected in cells expressing these pathogenic mutants, further supporting the functional relevance of ER-mitochondria contact sites for MFN2-related neurodegeneration.

In addition to CMT, links have been made between MFN2 dysregulation and both Parkinson’s and Alzheimer’s diseases (Han et al., 2011). Indeed, the frontal cortex of Alzheimer’s disease patients displays a reduction in MFN2 levels, as well as the hippocampal neurons of post-mortem patients (Wang et al., 2009; Manczak et al., 2011), which is recapitulated in Alzheimer’s disease models. In fact, production of amyloid β-peptide (aβ), the main component of amyloid plaques causative of Alzheimer’s disease, was found to be decreased upon knockdown of MFN2 (Leal et al., 2016). Furthermore, silencing of MFN2 led to an increase in ER-mitochondria contacts, characteristic of Alzheimer’s (Hedskog et al., 2013). However, contradictory results suggest that overexpression of MFN2 leads instead to reduction of aβ-mediated neuronal cell death (Park et al., 2015). In addition to ER-mitochondria contacts, a protective role of mitophagy has also been suggested (Kerr et al., 2017; Fang et al., 2019). Indeed, neurons affected in Alzheimer’s disease presented compromised mitophagy and accumulated defective mitochondria, consistent with the mitochondrial dysfunction characteristic of Alzheimer’s disease. Importantly, mitophagy induction diminished aβ as well as tau hyperphosphorylation, two hallmarks of Alzheimer’s disease (Fang et al., 2019). Supporting this idea, mitophagy induction prevented cognitive impairment in an Alzheimer’s disease mouse model and reversed memory impairment in both transgenic tau nematodes and mice (Fang et al., 2019). Regarding Parkinson’s disease, the link originates from MFN2 being a target of Parkin. First, loss of function mutations in the genes encoding for Parkin or PINK1 is found at the origin of early-onset PD (Kitada et al., 1998; Valente et al., 2004; Corti et al., 2011). In fact, Parkin mutations are primarily associated with autosomal recessive Parkinson’s disease and are the most known cause for this neuropathy. PD is characterized by progressive loss of dopaminergic neurons within substantia nigra, which attributes to Parkin an extremely important neuroprotective role. Second, the E3 ligase Parkin ubiquitylates MFN2 (Tanaka et al., 2010), and other OM proteins (Sarraf et al., 2013), being mitofusins rapidly degraded, among other Parkin targets (Chan et al., 2011; Sarraf et al., 2013). However, causative evidence for a role of MFN2-dependent mitophagy *via* PINK1/Parkin in Parkinson’s disease is still missing.

### Cardiac Dysfunction

A link between cardiac dysfunction and mitofusin impairment has been established over the years (Hall et al., 2014). First, ablation of mitofusins in cardiomyocytes, *Drosophila*, and mice caused cardiomyocyte dysfunction, rapid progressive dilated cardiomyopathy, and finally heart failure (Chen et al., 2011; Dorn et al., 2011; Papanicolaou et al., 2012; Song et al., 2017). Furthermore, heart phenotypes present in the fly were rescued upon expression of human mitofusins, supporting evolutionary conserved roles of mitofusins in the heart (Dorn et al., 2011). Interestingly, MFN2 was found to have an essential role in mice heart, in the metabolic shift from carbohydrates to fatty acids as the substrate preference, which occurs during perinatal period (Gong et al., 2015). An MFN2 mutant that cannot be phosphorylated by PINK1 and, therefore, inhibits Parkin-mediated mitophagy, prevented this mitochondrial metabolic maturation, and the respective hearts maintained the fetal metabolic signature (Gong et al., 2015). The authors suggested that mitochondria in fetal cardiomyocyte undergo MFN2-dependent mitophagy in order to allow their replacement by mature mitochondria. This places MFN2 as a central player in this essential metabolic shift. In fact, an active role of MFN2 for mitophagy of cardiac mitochondria had previously been suggested (Chen et al., 2011). Consistently, depletion of MFN1 and MFN2 in cardiomyocytes caused accumulation of defective mitochondria (Song et al., 2015, 2017). Moreover, no increase in mitophagy levels was observed, suggesting that ablation of both mitofusins interferes with a step in the mitophagic process required for the degradation of defective mitochondria (Song et al., 2015, 2017). In fact, despite the existence of fusion events in cardiac mitochondria (Weaver et al., 2014), they are discrete organelles rather than the continuous networks observed in other cell types, perhaps explaining the particular importance of mitophagy (Dorn, 2018).

### Type 2 Diabetes

A critical role of MFN2 for integrative physiology of whole body energy and glucose metabolism has been proposed, in neurons expressing orexigenic neuropeptide agouti-related protein (Agrp) and neurons expressing anorexigenic pro-opiomelanocortin (POMC; Ozcan, 2013; Dietrich et al., 2014; Schneeberger et al., 2014; Timper and Brüning, 2017). Moreover, MFN2 has been linked with diabetes and obesity, which are, *per se*, intimately related (Zorzano et al., 2009). MFN2 is found decreased in skeletal muscle from both obese and type 2 diabetic patients (Bach et al., 2005), in line with increased mitochondrial fission and reduced mitochondrial size, which are characteristic hallmarks of diabetes type 2 (Kelley et al., 2002; Toledo et al., 2006). Interestingly, body weight loss in obese subjects increased the expression of MFN2 in skeletal muscle and rescued mitochondrial size and number (Bach et al., 2005; Toledo et al., 2006). However, in adipose tissues of mice subjected to high-fat diet, the levels of MFN2 were increased, suggesting tissue-specific responses (Boutant et al., 2017). Moreover, ablation of MFN2 in adipocytes was beneficial, as it conferred better tolerance to glucose and protected against high-fat induced insulin resistance (Boutant et al., 2017; Mahdaviani et al., 2017). Interestingly, a concomitant decrease in mitochondria-lipid droplet interaction was observed, which decreased the lipolytic response of adipose tissues (Boutant et al., 2017). Lipid droplets are organelles present in adipose tissues responsible for the storage of lipid molecules. Upon specific metabolic conditions, these organelles release the lipid molecules, which can be further used for ATP production (Londos et al., 1999). Interestingly, the excessive storage of lipids in these organelles was shown to underlie metabolic disorders such as diabetes and obesity. These results suggest an interesting role of MFN2 in mitochondria-lipid droplets interaction, which had been previously described (Pidoux et al., 2011; Rambold et al., 2015). Consistently, lack of MFN2 in mice and *Drosophila* impairs lipid droplet formation and morphology (Sandoval et al., 2014; Mcfie et al., 2016). Interestingly, enzymes necessary for specific lipids biosynthesis commonly stored in lipid droplets are found enriched at MAM sites, where human MFN2 was also found to be enriched (De Brito and Scorrano, 2008). The nature of this relation is still poorly understood but might depend on a role of MFN2 in ER-mitochondria contacts, shown to be altered in different models of obesity and insulin resistance (Sebastián et al., 2012; Schneeberger et al., 2014; Filadi et al., 2017).

## Concluding Remarks

The importance of mitochondrial morphology for cellular fitness is increasingly clear. Moreover, the role of mitofusins as a response hub to different metabolic or stress states, by their selective recognition and modification has also been demonstrated. Ubiquitylation by E3 ligases and also phosphorylation or acetylation modulate a myriad of activating or repressing states in mitofusins. Further studies are required to elucidate controversial results and decipher the pathophysiological impact of mitofusin modifications in not only neurodegeneration and cardiac diseases but also energy expenditure-related diseases, such as obesity and diabetes.

## Author Contributions

Both authors wrote the manuscript. MJ drew the figures. ME-H coordinated the study.

### Conflict of Interest Statement

The authors declare that the research was conducted in the absence of any commercial or financial relationships that could be construed as a potential conflict of interest.
